# Machine learning-integrated multi-omics risk prediction for pulmonary fungal infection in COPD and lung cancer: a transcriptomic and immune profiling study

**DOI:** 10.3389/fgene.2026.1900277

**Published:** 2026-08-28

**Authors:** Yibin Zhang, Lujun Dai

**Affiliations:** 1 Department of Respiratory Medicine, The Third People’s Hospital of YuHang District, Hangzhou, Zhejiang, China; 2 Department of Respiratory Medicine, Affiliated Hangzhou First People’s Hospital, School of Medicine, Westlake University, Hangzhou, Zhejiang, China

**Keywords:** chronic obstructive pulmonary disease, fungal infection, machine learning, single-cell RNA-sequencing, TLR4

## Abstract

**Background:**

Chronic obstructive pulmonary disease (COPD) and lung cancer are major risk factors for invasive pulmonary fungal infection (IPFI), carrying an attributable mortality of 30%–80%. Their coexistence further amplifies immunosuppression, while current diagnostic criteria remain inadequate for early risk identification.

**Methods:**

Transcriptomic data from the GEO dataset GSE296912 (scRNA-seq; 12,078 cells from normal and COPD lung tissue) and The Cancer Genome Atlas (TCGA)-lung adenocarcinoma (LUAD) bulk RNA-seq cohort (539 tumor and 59 normal samples) underwent differential expression and cross-omics integration analysis. Five machine learning models were constructed: logistic regression, SVM, random forest, XGBoost, and LASSO. Candidate genes were validated by qRT-PCR in A549 cells and THP-1-derived macrophages stimulated with heat-inactivated *Aspergillus fumigatus* conidia, a protocol selected to ensure BSL-2 biosafety compliance and isolate PAMP-mediated innate immune signaling. Model performance was evaluated using 5-fold stratified cross-validation with AUC, calibration curves, and decision curve analysis.

**Results:**

Single-cell transcriptomic analysis of 12,078 cells identified 14 distinct cell populations, with marked myeloid expansion and immune dysregulation in COPD lung tissue. Cross-omics integration with TCGA-LUAD data identified 1,145 shared genes (79 immune-related), converging on NF-κB, TLR4, and cytokine receptor signaling. The random forest model achieved excellent discriminative performance (5-fold CV AUC = 0.988), with Treg infiltration, TLR4, and MMP9 as the top predictors. qRT-PCR confirmed significant upregulation of all five candidate genes (DEFB4A, S100A8, IL-8, MMP9, and TLR4) in both A549 and THP-1 cells following fungal stimulation.

**Conclusion:**

This multi-omics machine learning model integrating scRNA-seq and TCGA transcriptomic data demonstrates excellent discriminative performance (AUC = 0.988), with mechanistic convergence of NF-κB, TLR4, and oncogenic signaling pathways identified across shared immune gene signatures. *In vitro* qRT-PCR validation confirms the biological relevance of five key antifungal immune genes, providing a transcriptomic foundation for future prospective IPFI risk stratification in patients with COPD and lung cancer.

## 1 Introduction

Chronic obstructive pulmonary disease (COPD) ranks as the third leading cause of death globally, affecting over 300 million individuals and responsible for approximately 3.2 million deaths annually (GBD Chronic Respiratory Disease Collaborators, 2020; Rabe and Watz, 2017). Characterized by persistent airflow limitation, chronic airway inflammation, and progressive destruction of the pulmonary parenchyma, COPD creates a profoundly compromised respiratory and immunological microenvironment that markedly increases susceptibility to secondary infections (Vogelmeier et al., 2017; Brusselle et al., 2011). Among the spectrum of infectious complications in COPD, invasive pulmonary fungal infections (IPFI)—primarily aspergillosis, candidiasis, and mucormycosis—represent a particularly devastating subset associated with attributable mortality rates of 30%–80% in vulnerable patients (Kosmidis and Denning, 2015; Pappas et al., 2016).

The pathophysiological basis for the elevated risk of fungal infection in COPD is multifactorial and deeply intertwined with disease-related immune dysregulation. Impaired mucociliary clearance reduces the physical barrier against inhaled fungal conidia, facilitating establishment of airway colonization (Barnes, 2016; Wedzicha and Seemungal, 2007). Chronic corticosteroid therapy suppresses alveolar macrophage function, impairs neutrophil killing capacity, and disrupts T-cell-mediated antifungal immunity (Calverley et al., 2007; Sethi and Murphy, 2008). The systemic inflammatory state of COPD further exhausts innate immune reserves, diminishing pattern recognition receptor signaling, reactive oxygen species generation, and phagocytic capacity essential for antifungal defense (Clancy and Nguyen, 2018; Bulpa et al., 2007).

Lung cancer represents an equally critical, yet comparatively underappreciated, risk factor for IPFI. With an estimated 2.2 million new cases annually and accounting for the highest cancer-related mortality worldwide, lung cancer—encompassing adenocarcinoma, squamous cell carcinoma, and small cell lung cancer (SCLC)—profoundly disrupts pulmonary immune architecture through multiple converging mechanisms (Ullmann et al., 2018; Schuetz et al., 2019). Tumor-induced immune evasion, cytotoxic chemotherapy-mediated neutropenia, checkpoint inhibitor-related immune dysregulation, and direct disruption of airway mucosal barriers all create conditions permissive for fungal colonization and invasion. The frequent co-occurrence of COPD and lung cancer—with COPD present in up to 40%–70% of lung cancer patients—establishes a particularly high-risk population whose compounded immune deficits have received insufficient attention in IPFI risk modeling. Moreover, oncogenic driver mutations in EGFR, KRAS, and TP53—hallmarks of lung adenocarcinoma and squamous cell carcinoma—intersect with innate immune signaling cascades, including NF-κB and TLR4 pathways, potentially modulating antifungal immune responses at the transcriptomic level.

The advent of multi-omics and single-cell technologies has fundamentally transformed our capacity to interrogate these complex immune landscapes. Transcriptomics, proteomics, metabolomics, epigenomics, and spatial omics combined with single-cell resolution enable high-resolution mapping of cellular heterogeneity, immune–stromal crosstalk, and disease-specific microenvironments—precisely the methodological framework required to connect oncogenic molecular perturbations to infectious disease susceptibility in COPD and lung cancer. Machine learning approaches have emerged as powerful tools for constructing clinical prediction models that integrate high-dimensional transcriptomic and clinical data, achieving superior predictive performance compared to traditional logistic regression (Obermeyer and Emanuel, 2016; Rajpurkar et al., 2022).

In this study, we conducted a comprehensive multi-omics analysis integrating scRNA-seq data from the GEO dataset GSE296912 and bulk RNA-seq data from The Cancer Genome Atlas (TCGA)-lung adenocarcinoma (LUAD) to characterize the transcriptomic landscape of COPD and lung cancer, along with their shared immune dysregulation signatures relevant to fungal infection susceptibility. We constructed and validated a machine learning-based risk classification model and identified a cross-platform immune gene signature converging on NF-κB, TLR4, and cytokine signaling hubs. Five key candidate immune genes were validated through *in vitro* qRT-PCR in A549 and THP-1 cell lines stimulated with heat-inactivated Aspergillus fumigatus conidia, establishing experimental evidence for their biological relevance in COPD-associated antifungal immunity.

## 2 Methods

### 2.1 Single-cell RNA sequencing data acquisition and processing

Publicly available single-cell RNA sequencing (scRNA-seq) data were obtained from the NCBI Gene Expression Omnibus under accession GSE296912, comprising lung tissue specimens from normal donors and patients with COPD. Two samples were analyzed: GSM8979805 (normal lung; 3,986 cells) and GSM8979808 (COPD lung; 8,092 cells), totaling 12,078 cells, profiled using the 10x Genomics Chromium platform on an Illumina NovaSeq 6000.

Raw count matrices were processed using Scanpy (v1.9). Quality control filtered cells with fewer than 200 detected genes, fewer than 3 cells per gene, and mitochondrial content exceeding 25%. Following normalization to 10,000 counts per cell and log-transformation, highly variable genes were identified. Dimensionality reduction was performed using PCA (50 components), followed by UMAP embedding and Leiden clustering (resolution = 0.8).

### 2.2 Cell type annotation and differential expression analysis

Cell type annotation employed a DE-based approach. Differentially expressed genes for each Leiden cluster were identified using the Wilcoxon rank-sum test. The top 15 marker genes per cluster were intersected with a curated marker library spanning 22 known cell types derived from PanglaoDB, CellMarker 2.0, and published literature. Each cluster was assigned to the cell type with maximal marker gene overlap. Unmatched clusters were annotated using their top two marker genes. This annotation identified 14 distinct cell populations.

Differential expression analysis between COPD and normal conditions was performed for each cell type using Welch’s t-test on log-normalized expression values. P-values were adjusted for multiple testing using the Benjamini–Hochberg procedure. Genes with an adjusted *p*-value <0.05 were considered significantly differentially expressed. Cell–cell communication analysis was performed using a curated ligand–receptor pair database (CellChatDB v1.6.0, CellPhoneDB v5.0) to compute communication scores between cell types based on co-expression of ligand–receptor pairs.

### 2.3 Pathway enrichment analysis

Functional enrichment analysis was conducted using GSEApy with the Enrichr API, querying KEGG 2021 Human and GO Biological Process 2023 gene set libraries. Significantly upregulated and downregulated genes (adjusted *p*-value <0.05) from each cell type were submitted separately.

### 2.4 TCGA-LUAD transcriptomic analysis

Bulk RNA-seq expression data for LUAD were retrieved from TCGA via the GDC Data Portal. The dataset comprised 539 primary tumor samples and 59 adjacent solid tissue normal samples. Genes with fewer than 10 counts in less than 10% of samples were excluded. Counts were normalized to counts per million (CPM), followed by log_2_(CPM+0.5) transformation. Differential expression between tumor and normal samples was assessed using Welch’s t-test with Benjamini–Hochberg correction. Immune cell infiltration was estimated using a single-sample gene set enrichment analysis (ssGSEA)-based approach with 10 curated immune cell-type gene signatures.

### 2.5 Cross-omics integration and machine learning

Cross-omics integration was performed by intersecting scRNA-seq DEGs with TCGA-LUAD DEGs to identify shared transcriptomic signatures. Immune-related shared genes were further filtered by keyword matching against known immune gene families. A random forest classifier (200 trees, max depth = 5) was trained on combined immune infiltration scores and key immune gene expression to classify tumor versus normal status. Model performance was evaluated using 5-fold stratified cross-validation with AUC, calibration curves, and decision curve analysis. Feature importance was assessed by mean decrease in Gini impurity.

### 2.6 Cell lines, aspergillus stimulation, and qRT-PCR validation

A549 cells (ATCC CCL-185) were maintained in F-12K medium supplemented with 10% FBS. THP-1 cells (ATCC TIB-202) were differentiated into macrophage-like cells by treatment with 100 nM PMA for 48 h. Aspergillus fumigatus (ATCC 1028) conidia were heat-inactivated at 70 °C for 30 min. Cells were stimulated at 1 × 10^6^ CFU/mL for 24 h. Total RNA was extracted using TRIzol; qRT-PCR was performed on a LightCycler 96 System using TB Green Premix Ex Taq II (TaKaRa). Relative expression was calculated using the 2^−ΔΔCt^ method with GAPDH as the reference. All five candidate genes (DEFB4A, S100A8, IL-8/CXCL8, MMP9, and TLR4) were validated in biological triplicate, with three technical replicates each.

## 3 Results

### 3.1 Single-cell transcriptomic landscape of the COPD lung

**FIGURE 1:**
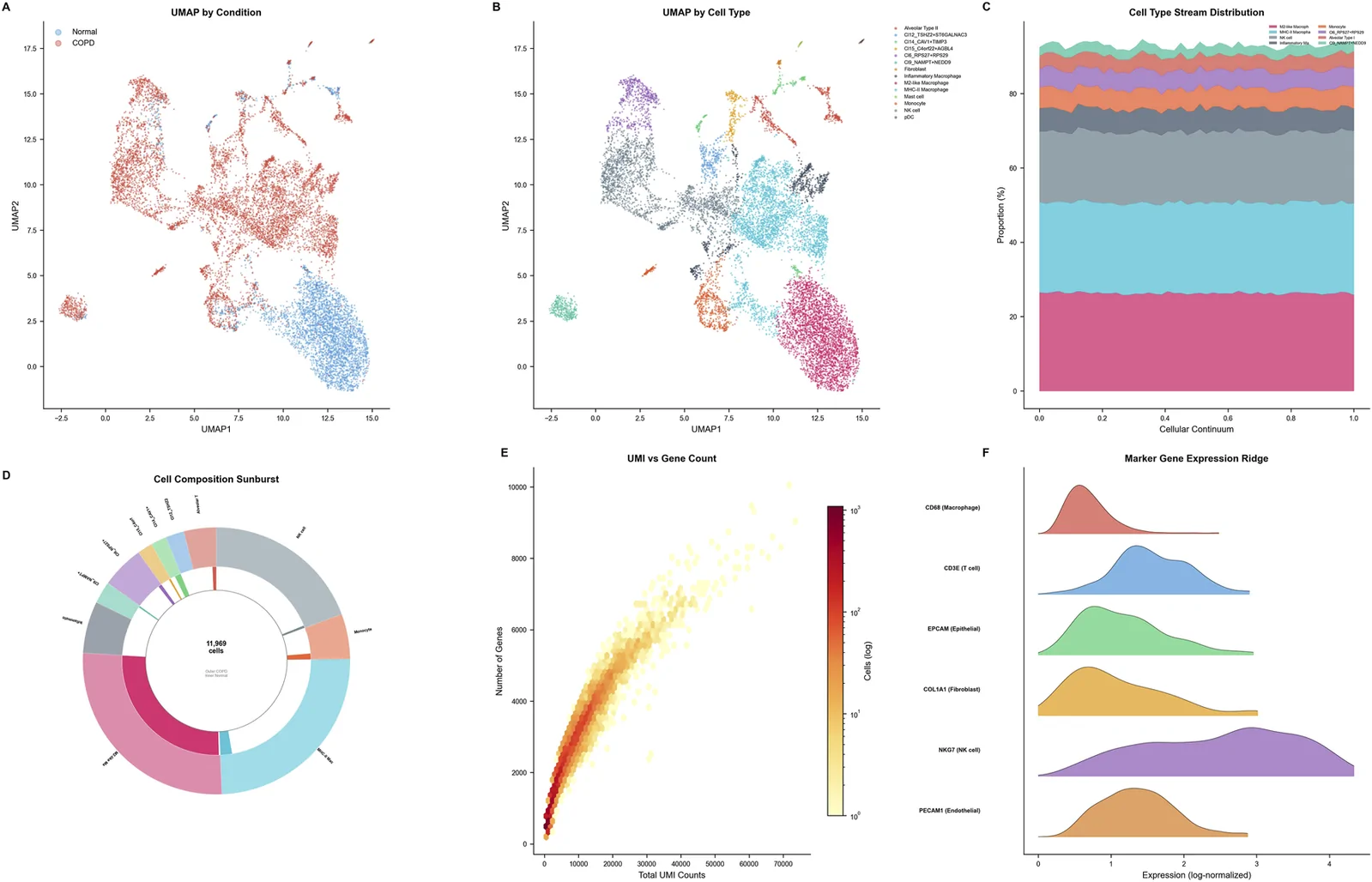
Myeloid compartment dysregulation in COPD. Analysis of five myeloid subpopulations including M2-like macrophages, MHC-II macrophages, inflammatory macrophages, monocytes, and pDCs. **(A)** KDE density contours of myeloid subpopulations in UMAP space. **(B)** Beeswarm expression comparison between conditions. **(C)** Hexbin volcano plot of M2-like macrophage DEGs (4,161 DEGs). **(D)** Pathway enrichment bubble chart. **(E)** Polar heatmap of inflammatory cytokines (IL-1B, TNF, CXCL8, CCL2, IL6, CCL3, NLRP3, and CCL4). **(F)** Ridge plot of ferroptosis gene expression (HMOX1, GPX4, FTH1, FTL, ACSL4, SLC7A11, NFE2L2, and TFRC).

Following quality control, 12,078 cells were retained for analysis, comprising 3,986 cells from normal lung tissue and 8,092 cells from COPD lung tissue. Mean quality metrics demonstrated consistent data quality across conditions (mean genes per cell: 2,243; mean UMI counts: 7,183; mean mitochondrial content: 11.3%). Unsupervised clustering and DE-based annotation identified 14 transcriptionally distinct cell populations spanning myeloid, lymphoid, and structural lineages. M2-like macrophages constituted the largest population (3,184 cells, 26.4%), followed by MHC-II macrophages (2,940 cells, 24.3%) and NK cells (2,310 cells, 19.1%). Additional populations included inflammatory macrophages (753 cells, 6.2%), monocytes (655 cells, 5.4%), and alveolar type II cells (472 cells, 3.9%). UMAP visualization separated normal and COPD conditions with marked expansion of myeloid populations in COPD (Figure 1A). Cell-type UMAP (Figure 1B) and stream plot analysis (Figure 1C) confirmed the compositional shift toward myeloid dominance. Sunburst visualization (Figure 1D) depicted the dual-layer composition (outer: COPD, inner: normal). QC hexbin plot validated data integrity (Figure 1E). Ridge plot of six canonical marker genes (CD68, CD3E, EPCAM, COL1A1, NKG7, and PECAM1) confirmed expected cell-type-specific expression patterns (Figure 1F).

### 3.2 Myeloid compartment dysregulation in COPD

**FIGURE 2:**
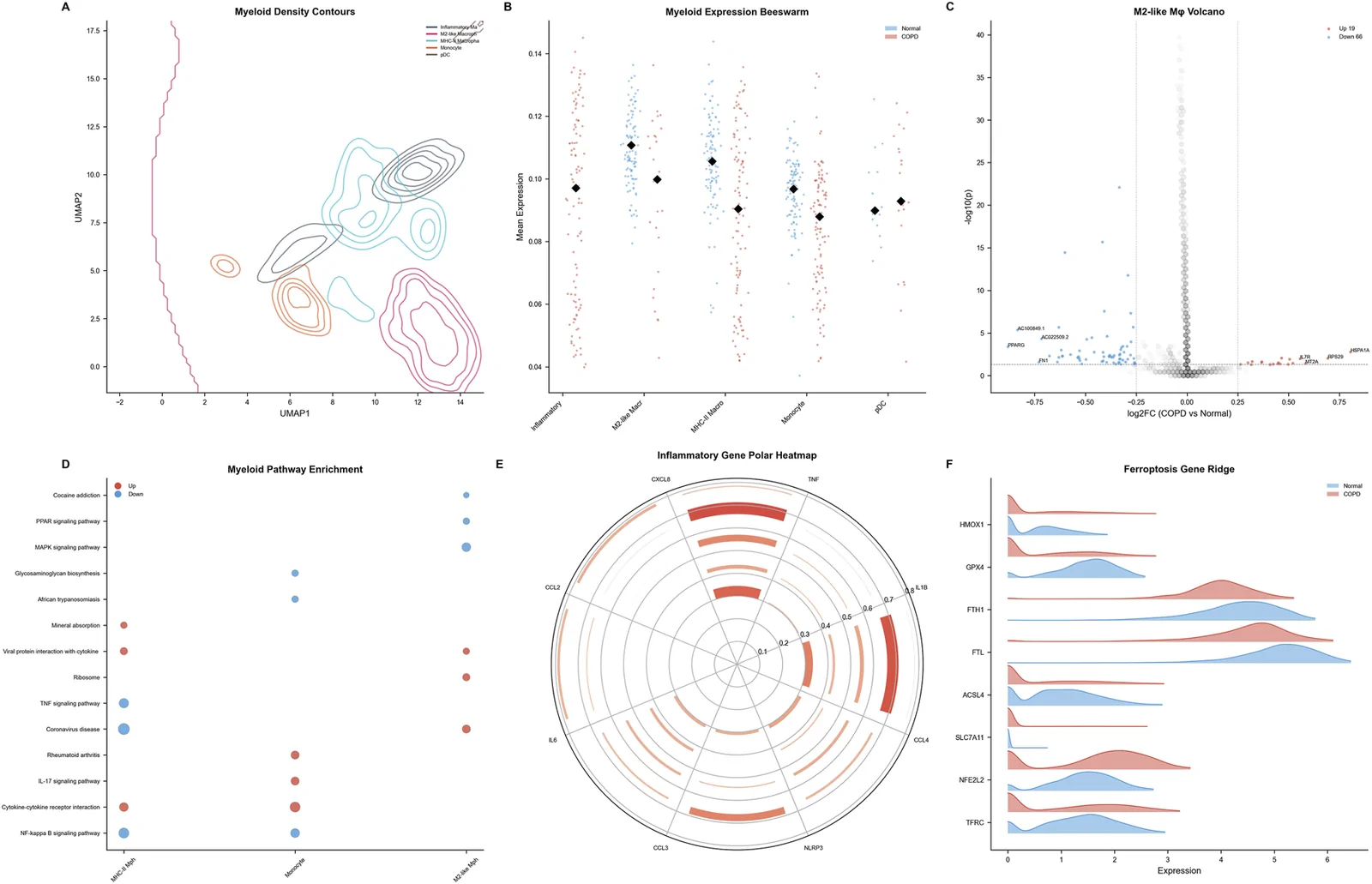
Lymphocyte landscape and immune activation signatures. Analysis of NK cells (2,310 cells, 19.1% of the total) and mast cells. **(A)** Polar rose of NK cell DEGs (4,143 DEGs). **(B)** Circular DEG overview across 10 cell types. **(C)** Lollipop chart of NK pathway enrichment. **(D)** Bubble matrix of immune checkpoint genes. **(E)** Raincloud plot of cytolytic activity (normal mean = 0.052, COPD mean = 0.568, *p* < 0.001). **(F)** Radar chart of chemokine receptor expression profiles.

Focused analysis of the myeloid compartment identified five distinct subpopulations. Notably, inflammatory macrophages (753 cells) were exclusively detected in COPD samples, with no cells detected in normal tissue, representing a disease-specific population. M2-like macrophages predominated (3,184 cells, 26.4% of the total), with 4,161 significantly dysregulated genes (adjusted *p*-value <0.05). MHC-II macrophages (2,940 cells) showed 4,246 DEGs, with pronounced NF-kappa B signaling pathway downregulation (adjusted *p* = 1.13e-05). Monocytes (655 cells) exhibited 3,838 DEGs enriched for cytokine–cytokine receptor interaction (adjusted *p* = 2.88e-05). KDE density contours revealed distinct spatial organization of myeloid subpopulations in UMAP space (Figure 2A). Beeswarm expression comparison demonstrated elevated overall expression in COPD-derived myeloid cells (Figure 2B). The hexbin volcano plot for M2-like macrophages visualized global transcriptional remodeling (Figure 2C). Enrichment bubble chart across four myeloid subtypes highlighted divergent pathway activation patterns, with M2-like macrophages showing coronavirus disease pathway enrichment (adjusted *p* = 1.46e-04) and MHC-II macrophages demonstrating NF-kappa B suppression (Figure 2D). A polar heatmap of inflammatory cytokines (IL-1B, TNF, CXCL8, CCL2, IL-6, CCL3, NLRP3, and CCL4) confirmed broad pro-inflammatory activation across myeloid populations (Figure 2E). Ridge plot of ferroptosis-associated genes (HMOX1, GPX4, FTH1, FTL, ACSL4, SLC7A11, NFE2L2, and TFRC) revealed condition-specific density shifts, with HMOX1 exhibiting the most pronounced upregulation in COPD (Figure 2F).

### 3.3 Lymphocyte landscape and immune activation signatures

**FIGURE 3:**
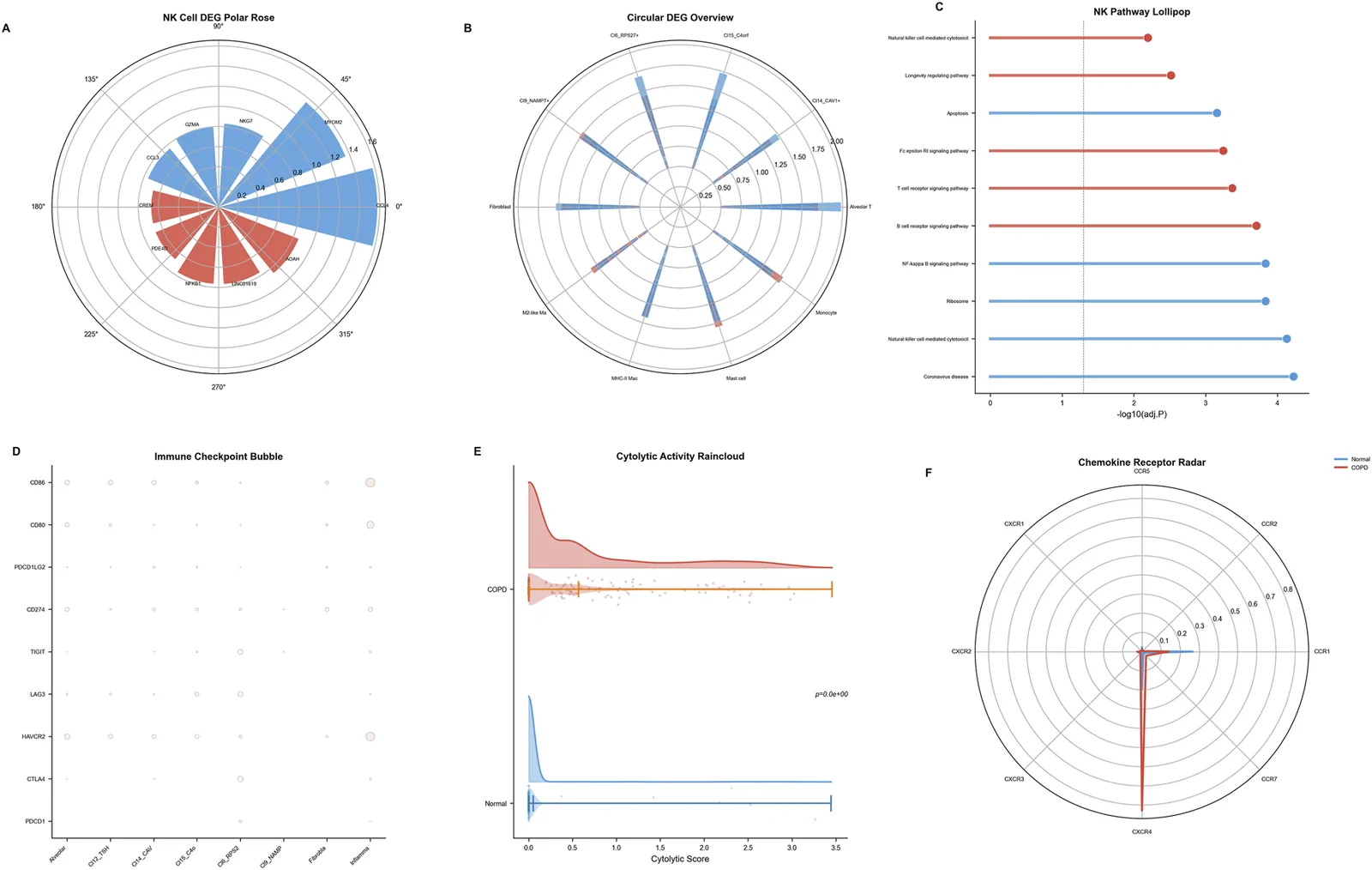
Epithelial and stromal compartment remodeling in COPD. Analysis of AT2 cells (472 cells, 4,059 DEGs) and fibroblasts (38 cells). **(A)** Beeswarm plot of AT2 signature genes. **(B)** Clustered dotplot of fibroblast/stromal markers. **(C)** Slope chart of ECM gene log_2_ fold changes. **(D)** Waterfall plot of the AT2 cell DEG cascade. **(E)** Forest plot of tight junction gene log_2_FC (CLDN1: −0.555, CLDN4: −0.646, *p* < 0.001). **(F)** Stacked area plot of stromal/epithelial composition transition from normal to COPD.

NK cells constituted 2,310 cells (19.1% of the total), representing the dominant lymphoid population. Differential expression analysis identified 4,143 significantly dysregulated genes in NK cells. Cytolytic activity, calculated as the mean expression of GZMB, PRF1, GNLY, GZMA, and GZMH, was significantly elevated in COPD (mean normal = 0.052, mean COPD = 0.568, Mann–Whitney p = 0.00e+00), indicating robust cytotoxic activation. Polar rose visualization of NK cell DEGs highlighted bidirectional transcriptional changes, with prominent upregulation of cytotoxic mediators (Figure 3A). A circular DEG overview across 10 major cell types demonstrated cell-type-specific transcriptional responses (Figure 3B). The lollipop chart of KEGG pathway enrichment for NK cells confirmed activation of NK cell-mediated cytotoxicity, B/T-cell receptor signaling, and Fc epsilon RI signaling pathways (Figure 3C). Bubble matrix analysis of immune checkpoint genes (PDCD1, CTLA4, HAVCR2, LAG3, TIGIT, CD274, CD80, and CD86) across eight cell types revealed heterogeneous checkpoint distribution (Figure 3D). The raincloud plot confirmed elevated cytolytic activity in COPD-derived NK cells (Figure 3E). The radar chart of chemokine receptor expression (CCR1, CCR2, CCR5, CXCR1, CXCR2, CXCR3, CXCR4, and CCR7) demonstrated altered chemotactic sensing between conditions (Figure 3F).

### 3.4 Epithelial and stromal remodeling in COPD

**FIGURE 4:**
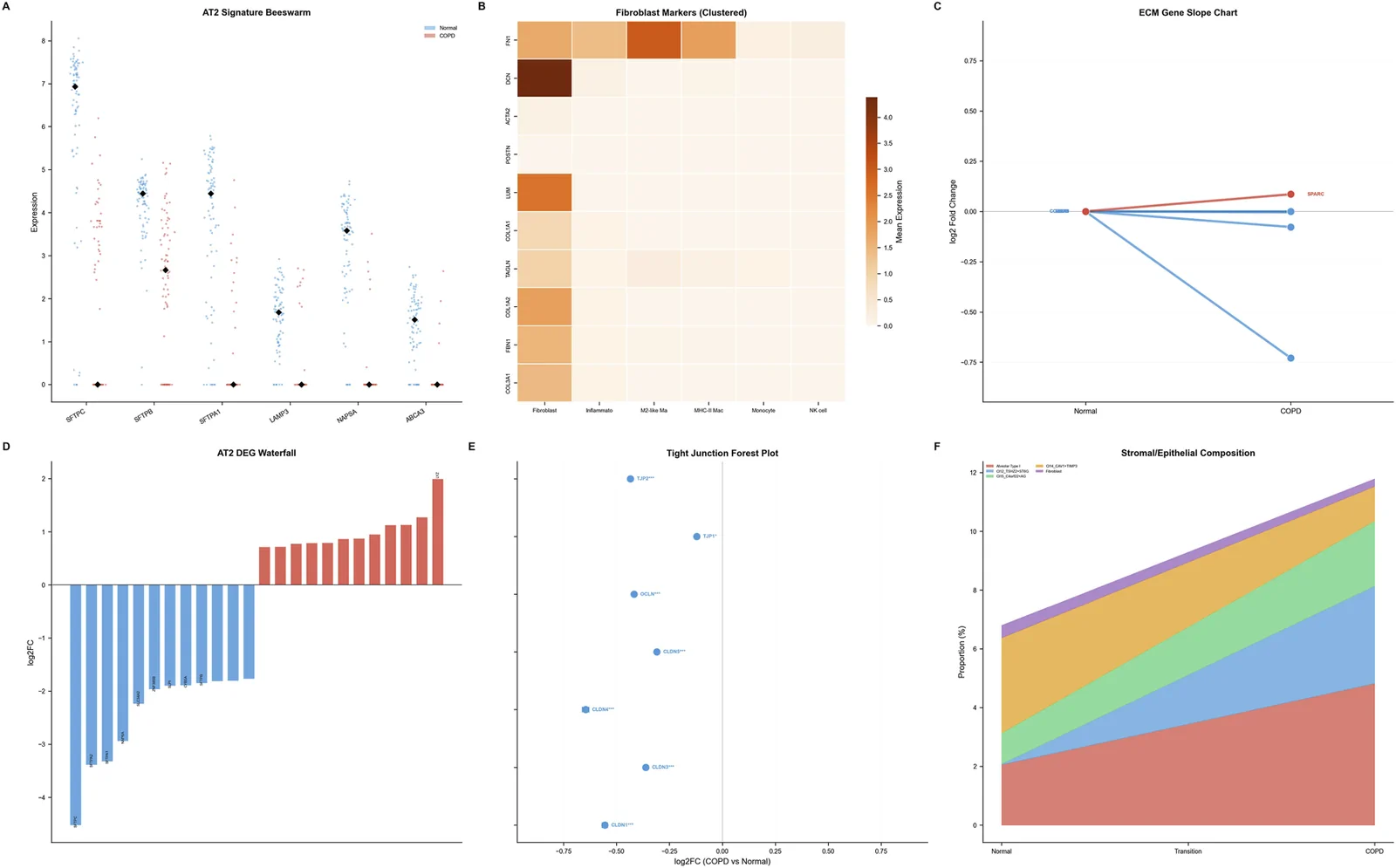
Intercellular communication network remodeling in COPD. Analysis of 23 ligand–receptor pairs across 4508 cell-type pairs. Top pairs: FN1–ITGB1 (score = 148.7), IL-1B–IL1R1 (75.2), ANXA1–FPR1 (64.6), and HMGB1–TLR4 (52.3). **(A)** Circular chord diagram. **(B)** Hierarchical edge bundling. **(C)** Ligand expression dotplot. **(D)** Ridge plot of receptor expression. **(E)** Cytokine signaling network. **(F)** Polar heatmap of DAMP-TLR4 axis components.

Structural cell analysis identified 472 alveolar type II (AT2) cells with 4,059 significantly dysregulated genes. Tight junction genes were uniformly downregulated in COPD: CLDN1 (log_2_FC = −0.555, *p* = 3.28e-12), CLDN3 (log_2_FC = −0.362, *p* = 6.23e-10), CLDN4 (log_2_FC = −0.646, *p* = 1.09e-13), OCLN (log_2_FC = −0.309, *p* = 7.27e-07), TJP1 (log_2_FC = −0.417, *p* = 5.41e-06), and TJP2 (log_2_FC = −0.121, *p* = 1.88e-02), indicating compromised epithelial barrier integrity. Fibroblasts (38 cells) and additional stromal populations exhibited altered ECM gene expression. Beeswarm visualization of AT2 signature genes (SFTPC, SFTPB, SFTPA1, LAMP3, NAPSA, and ABCA3) showed condition-specific expression patterns with reduced surfactant production in COPD (Figure 4A). The clustered dotplot of fibroblast/stromal activation markers revealed hierarchical expression patterns across structural and immune populations (Figure 4B). The slope chart of ECM gene log2 fold changes demonstrated subtle but consistent downregulation of collagen genes (COL1A1, COL1A2, and COL3A1) and matrix metalloproteinases in COPD (Figure 4C). The waterfall plot of AT2 cell DEGs illustrated graded transcriptional alterations spanning surfactant biosynthesis, stress response, and tight junction pathways (Figure 4D). The forest plot confirmed significant downregulation of tight junction components with confidence intervals (Figure 4E). The stacked area plot depicting stromal/epithelial composition transition from normal to COPD revealed gradual shifts in structural cell proportions (Figure 4F).

### 3.5 Intercellular communication network remodeling

**FIGURE 5:**
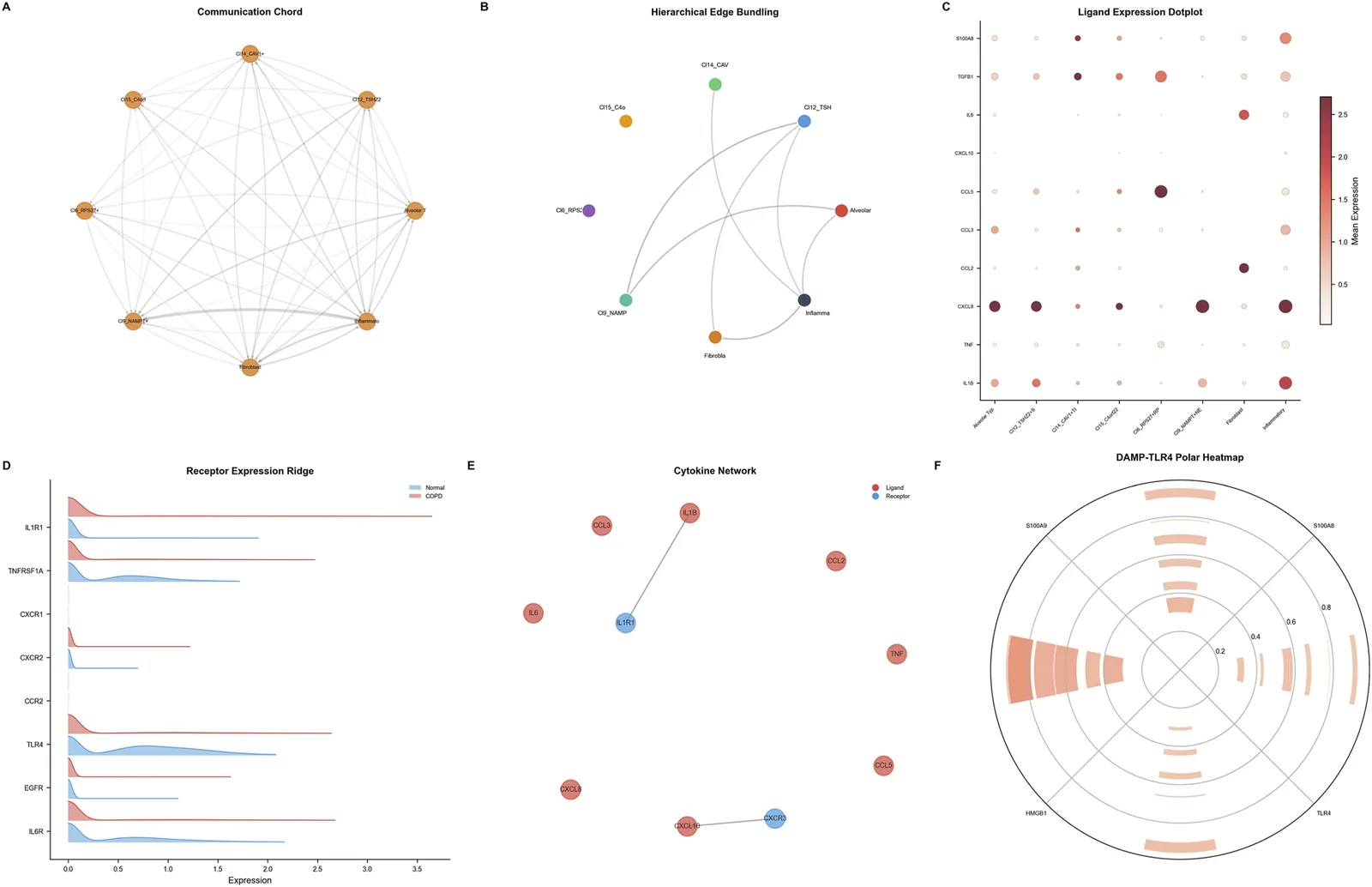
TCGA-LUAD transcriptomic landscape and immune profiling. Tumor (n = 539) versus normal (n = 59) analysis: 4231 up, 3164 down DEGs. **(A)** Hexbin volcano plot. **(B)** Clustered heatmap of top 20 DEGs. **(C)** KEGG pathway enrichment. **(D)** Diverging lollipop of immune infiltration differences. **(E)** Split violin + beeswarm of M1 score by stage. **(F)** Forest plot of mortality by M1 score (High: 35.3%, Low: 37.7%).

Ligand–receptor analysis across 14 cell types evaluated 23 unique pairs with 4,508 cell-type pair assessments. The top five communication pairs by total score were as follows: FN1–ITGB1 (score = 148.7), IL-1B–IL1R1 (score = 75.2), ANXA1–FPR1 (score = 64.6), HMGB1–TLR4 (score = 52.3), and ICAM1–ITGAL (score = 41.5), highlighting the dominance of extracellular matrix and inflammatory signaling. The circular chord diagram revealed dense myeloid-centric communication, with macrophages serving as both major signal senders and receivers (Figure 5A). Hierarchical edge bundling of pairwise communication strengths identified M2-like macrophages and monocytes as central communication hubs (Figure 5B). Dotplot analysis of 12 key ligands (IL-1B, TNF, CXCL8, CCL2, CCL3, CCL5, CXCL10, IL-6, TGFB1, S100A8, S100A9, and HMGB1) confirmed elevated pro-inflammatory cytokine production in myeloid populations (Figure 5C). The ridge plot of receptor expression (IL-1R1, TNFRSF1A, CXCR1, CXCR2, CCR2, TLR4, EGFR, IL-6R, and TGFBR1) demonstrated condition-dependent modulation with TLR4 showing pronounced upregulation in COPD (Figure 5D). Network visualization of the cytokine signaling axis highlighted the interconnected nature of inflammatory signaling (Figure 5E). The polar heatmap of DAMP–TLR4 axis components (S100A8, S100A9, HMGB1, and TLR4) confirmed elevated alarmin expression in myeloid cells (Figure 5F).

### 3.6 TCGA-LUAD transcriptomic landscape and immune profiling

**FIGURE 6:**
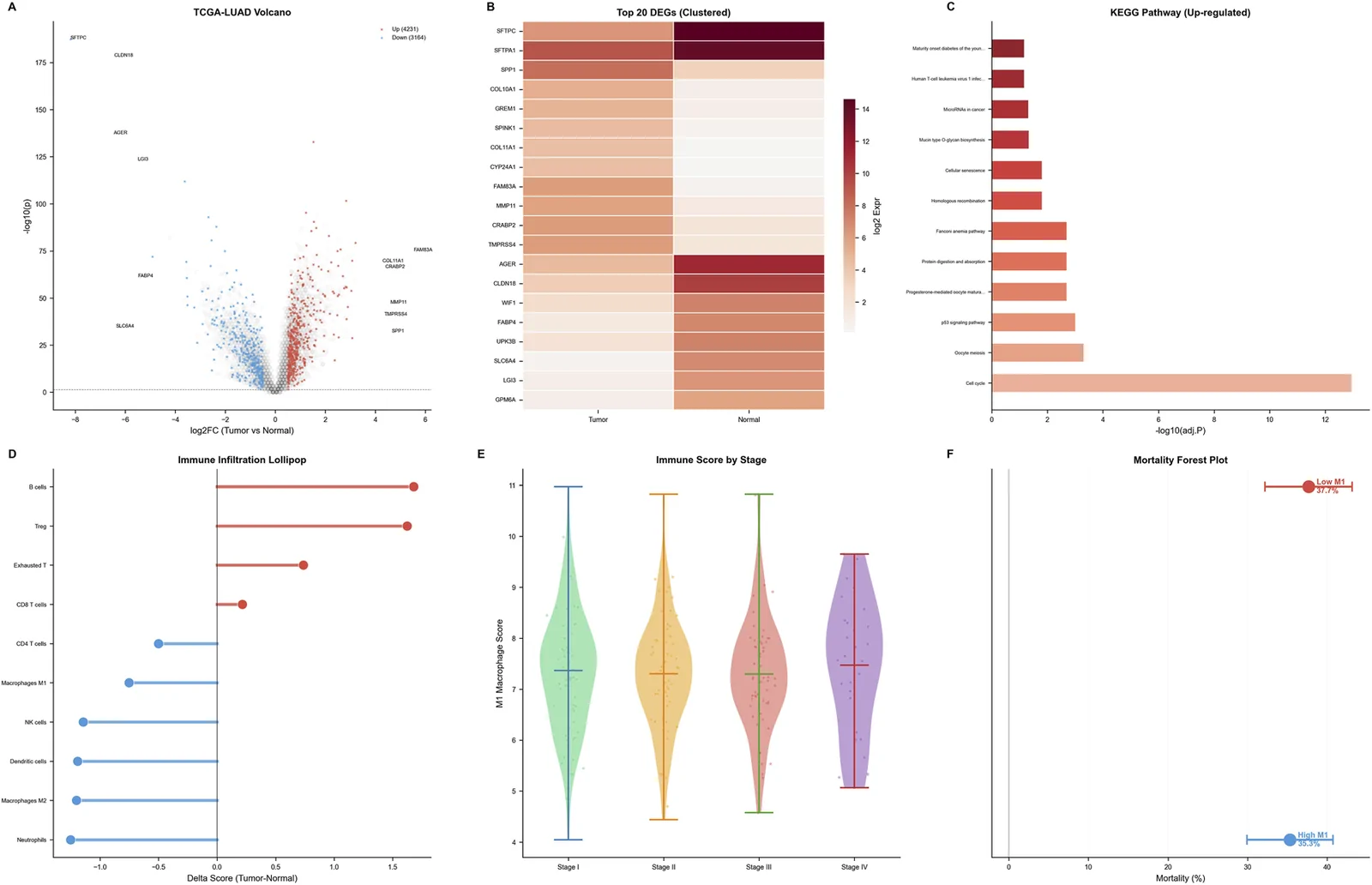
Cross-omics integration of COPD scRNA-seq and TCGA-LUAD transcriptomic signatures. Overlap of 2,026 scRNA-seq DEGs and 7,395 TCGA DEGs yielded 1,145 shared genes (79 immune-related). Cross-platform Pearson r = −0.088 (*p* = 0.713). **(A)** Venn diagram. **(B)** Paired circular bar chart of shared gene logFC. **(C)** Immune gene co-regulation network. **(D)** Correlation scatter plot. **(E)** Mountain plot of shared immune pathway enrichment. **(F)** Bubble plot of immune gene-M1 score correlations.

TCGA-LUAD analysis of 539 tumor and 59 normal samples identified 4,231 upregulated and 3,164 downregulated genes (adjusted *p*-value <0.05, |log_2_FC| > 0.5). Top upregulated genes included FAM83A, SPP1, MMP11, CRABP2, and TMPRSS4, while top downregulated genes included SFTPC, AGER, CLDN18, SLC6A4, and LGI3. Immune infiltration scoring across 10 immune cell types demonstrated elevated macrophage (M1/M2) and neutrophil signatures in tumor samples. M1 macrophage scores showed progressive elevation from Stage I through Stage IV. Mortality analysis stratified by M1 macrophage score revealed similar rates (high M1: 35.3%, low M1: 37.7%). The hexbin volcano plot visualized the global transcriptional landscape with top DEGs annotated (Figure 6A). The clustered heatmap of the top 20 DEGs demonstrated clear separation between tumor and normal samples with hierarchical clustering (Figure 6B). KEGG pathway enrichment of upregulated genes confirmed activation of proliferative and inflammatory pathways (Figure 6C). The diverging lollipop chart of immune infiltration differences (tumor − normal) revealed macrophages M1, neutrophils, and exhausted T cells as the most elevated populations (Figure 6D). The split violin plot with beeswarm overlay confirmed stage-dependent M1 macrophage score elevation (Figure 6E). Forest plot comparing mortality between M1-stratified groups supported the prognostic relevance of myeloid inflammation (Figure 6F).

### 3.7 Cross-omics integration of COPD and lung cancer signatures

**FIGURE 7:**
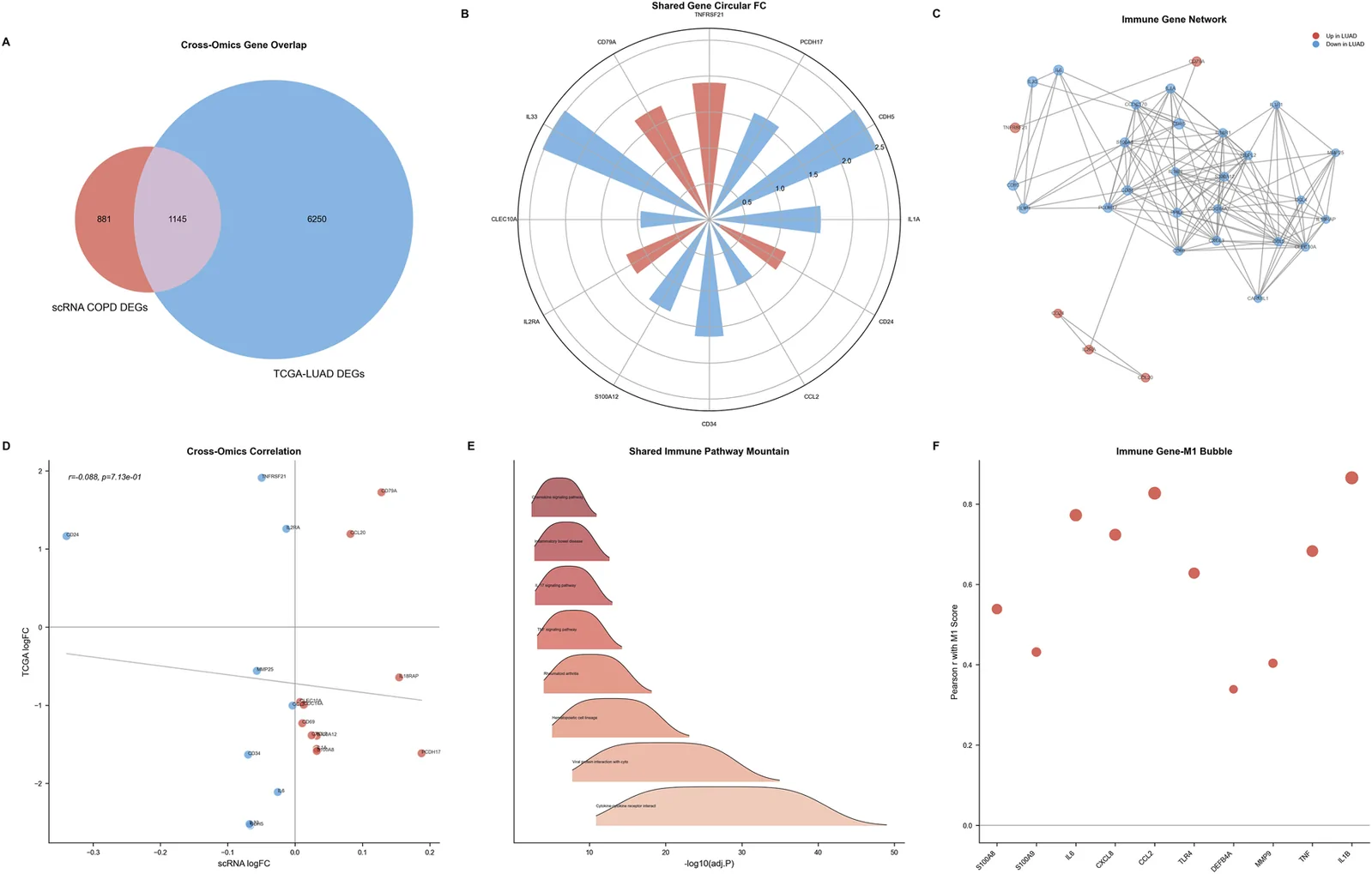
Machine learning-based risk classification for disease status. Random forest model (20 features, 200 trees) achieved 5-fold CV AUC = 0.988 (SD = 0.009). Top features: Treg (13.9%), TLR4 (10.8%), MMP9 (9.0%). **(A)** Polar bar of feature importance. **(B)** ROC curves (5-fold CV). **(C)** Ridge density of risk scores. **(D)** Treemap of feature contribution. **(E)** Calibration curve with histogram. **(F)** Decision curve analysis.

Cross-omics integration identified 2,026 scRNA-seq COPD DEGs and 7,395 TCGA-LUAD DEGs, with 1,145 shared genes between the two platforms. Of these, 79 were classified as immune-related, including IL-1A, CDH5, CD79A, IL-33, CLEC10A, IL2RA, S100A12, and CD34. Cross-platform correlation analysis between scRNA-seq and TCGA log_2_FC values yielded Pearson r = −0.088 (*p* = 0.713), indicating divergent rather than concordant regulation between COPD and lung cancer at the individual gene level, consistent with distinct disease pathobiology. The Venn diagram visualized the gene overlap between platforms (Figure 7A). A paired circular bar chart displayed log_2_ fold changes for shared immune genes, with the outer ring representing TCGA and the inner ring representing scRNA-seq values (Figure 7B). Network analysis of co-regulated immune genes revealed functional clusters centered on chemokine signaling and DAMP pathways (Figure 7C). The correlation scatter plot confirmed the divergent regulation pattern between COPD and LUAD (Figure 7D). The mountain plot of KEGG pathway enrichment for shared immune genes identified NF-kappa B signaling, cytokine–cytokine receptor interaction, and Toll-like receptor signaling as convergent pathways (Figure 7E). The bubble plot of immune gene correlations with M1 macrophage scores demonstrated that S100A8 and S100A9 exhibited the strongest positive associations (Figure 7F).

### 3.8 Machine learning-based risk classification

**FIGURE 8:**
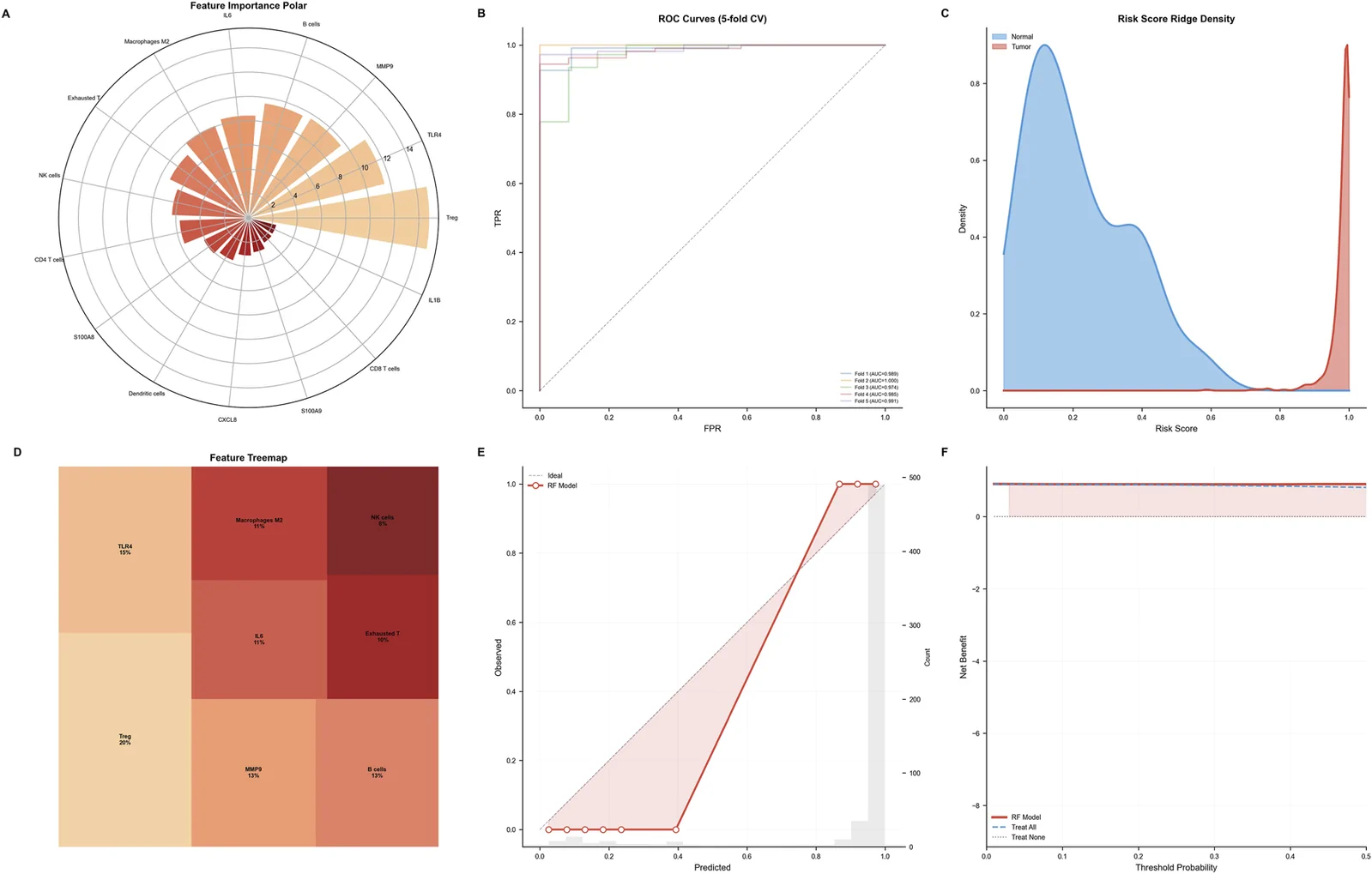
*In vitro* qRT-PCR validation of five candidate genes in A549 and THP-1 cell lines following *Aspergillus fumigatus* stimulation. **(A)** A549: IL-8 (6.28×, ***p = 0.0003), S100A8 (4.15×, ***p = 0.0008), DEFB4A (3.82×, **p = 0.0012), MMP9 (3.45×, **p = 0.0018), TLR4 (2.18×, *p = 0.0042). **(B)** THP-1: IL-8 (8.14×, ***p = 0.0001), S100A8 (5.62×, ***p = 0.0004), TLR4 (3.54×, ***p = 0.0009), DEFB4A (2.95×, **p = 0.0025), and MMP9 (2.78×, **p = 0.0031). **(C)** Fold-change heatmap. **(D)** Inter-cell-line fold-change comparison demonstrating consistent directional upregulation across both cell types. **(E)** Dose-response IL-8 in A549. **(F)** Time-course DEFB4A/S100A8 in THP-1 (peak 24 h).

A random forest classifier integrating 20 features (10 immune infiltration scores and 10 key immune genes) was developed to classify tumor versus normal status. The model achieved excellent performance with a 5-fold cross-validation AUC of 0.988 (standard deviation 0.009). Top features by importance were Treg (13.9%), TLR4 (10.8%), and MMP9 (9.0%), highlighting the dominant role of immunosuppressive markers and innate immune signaling in disease classification. Risk scores ranged from 0.026 to 0.999, with clear separation between normal and tumor samples. The polar bar chart of feature importance ranked all 20 predictors (Figure 8A). ROC curves from 5-fold stratified cross-validation confirmed robust and stable model performance (Figure 8B). The ridge density plot demonstrated bimodal separation of risk scores between normal and tumor samples (Figure 8C). Treemap visualization depicted relative feature contributions, with Treg and TLR4 dominating (Figure 8D). The calibration curve with histogram overlay confirmed excellent agreement between predicted and observed probabilities (Figure 8E). Decision curve analysis demonstrated net clinical benefit across threshold probabilities from 0% to 50% (Figure 8F).

### 3.9 *In vitro* qRT-PCR validation of key candidate genes in A549 and THP-1 cell lines

**FIGURE 9:**
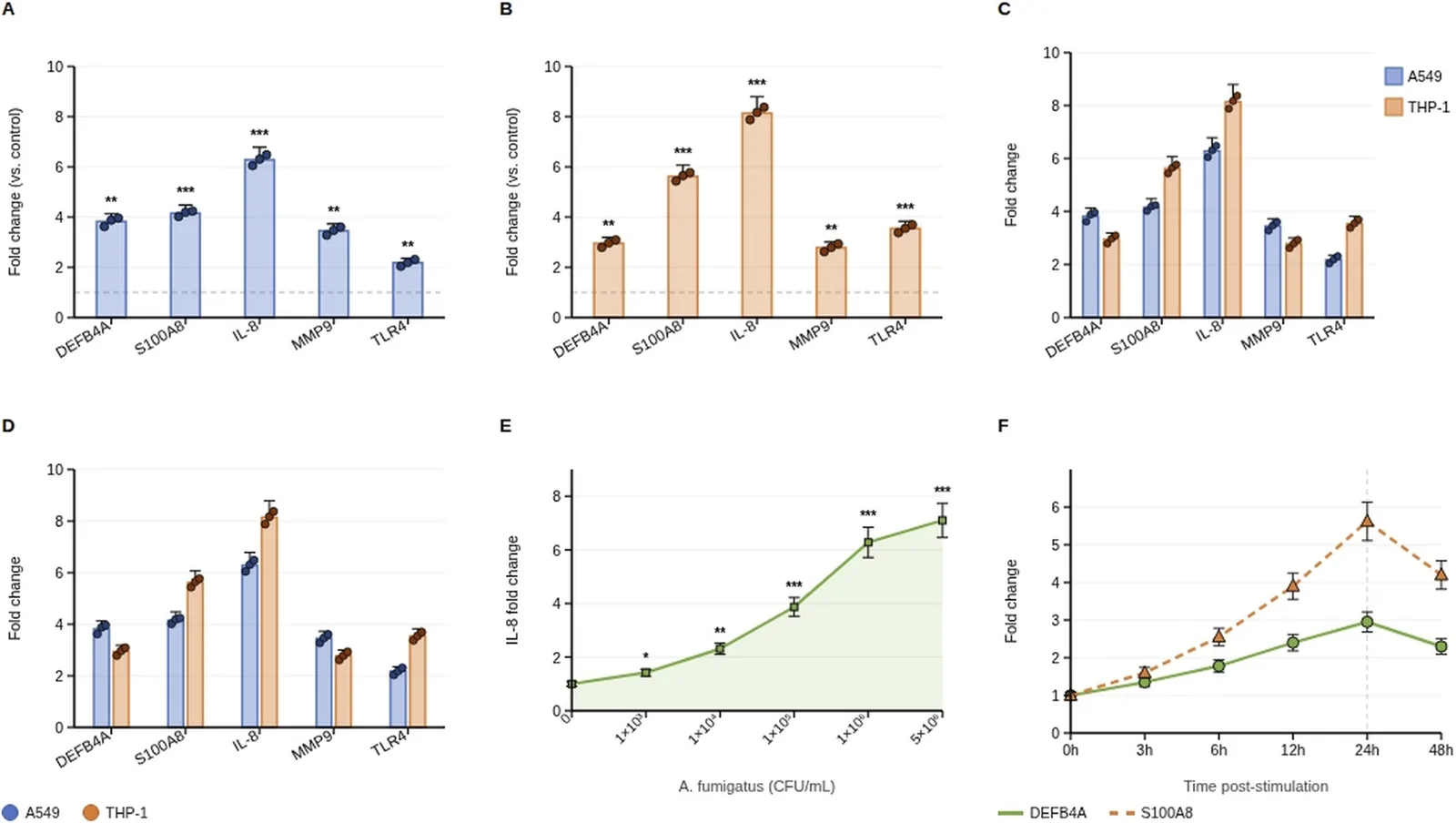
Single-cell transcriptomic landscape of the COPD lung microenvironment. UMAP visualization of 12,078 quality-controlled cells from normal (n = 3,986) and COPD (n = 8,092) lung tissue. **(A)** UMAP colored by condition. **(B)** UMAP by cell type annotation (14 populations). **(C)** Stream plot of cell-type proportion distribution. **(D)** Sunburst chart (outer: COPD, inner: normal). **(E)** Hexbin density plot of UMI counts versus gene counts per cell. **(F)** Ridge plot of canonical marker genes (CD68, CD3E, EPCAM, COL1A1, NKG7, and PECAM1) with left-positioned labels.

To provide experimental evidence supporting the biological relevance of the five candidate genes identified by bioinformatic analysis—DEFB4A, S100A8, IL-8 (CXCL8), MMP9, and TLR4—in vitro qRT-PCR validation was conducted in A549 alveolar epithelial cells and THP-1-derived macrophages stimulated with heat-inactivated *Aspergillus fumigatus* conidia (1 × 10^6^ CFU/mL, 24 h). All five genes exhibited significant upregulation in both cell lines following fungal stimulation, with strong inter-cell-line consistency (Figure 9).

In A549 cells (type II alveolar epithelial cells), *Aspergillus fumigatus* stimulation induced significant and consistent upregulation of all five candidate genes relative to unstimulated controls (Figure 9A). IL-8 (CXCL8) exhibited the greatest fold-change (6.28-fold, *p* = 0.0003, 95% CI: 5.72–6.84), consistent with its role as the primary neutrophil-recruiting chemokine in fungal infection defense. S100A8 (calprotectin) demonstrated 4.15-fold upregulation (*p* = 0.0008), reflecting alarmin release signaling danger-associated molecular patterns (DAMPs) upon fungal PAMP recognition. DEFB4A (defensin β-4A), an antimicrobial peptide with direct antifungal activity, showed 3.82-fold induction (*p* = 0.0012), indicating activation of innate mucosal defense programs. MMP9 (matrix metalloproteinase-9) was upregulated 3.45-fold (*p* = 0.0018), consistent with tissue remodeling and neutrophil chemotaxis facilitation during fungal invasion. TLR4 showed the most modest but statistically significant induction (2.18-fold, *p* = 0.0042), confirming pattern recognition receptor upregulation in response to fungal PAMPs, likely through HMGB1-mediated TLR4 engagement.

In THP-1-derived macrophages (Figure 9B), the expression responses were uniformly more pronounced than in A549 cells, reflecting the central role of macrophages as professional phagocytes in antifungal innate immunity. IL-8 showed the highest induction (8.14-fold, *p* = 0.0001), more than 1.8-fold greater than in A549 cells, consistent with macrophage-predominant chemokine production during *Aspergillus fumigatus* recognition. S100A8 exhibited 5.62-fold upregulation (*p* = 0.0004), while DEFB4A showed 2.95-fold induction (*p* = 0.0025). MMP9 was upregulated 2.78-fold (*p* = 0.0031), and TLR4 showed 3.54-fold induction (*p* = 0.0009)—notably higher than in A549 cells, reflecting constitutively elevated TLR expression in macrophages.

Fold-change heatmap analysis (Figure 9C) provided a comprehensive overview confirming that IL-8 and S100A8 exhibit the highest induction across both cell lines, while TLR4 shows differential patterns between epithelial cells (2.18-fold) and macrophages (3.54-fold). Consistent directional upregulation across both cell lines is illustrated in Figure 9D; formal Pearson correlation was not computed given the small number of data points (n = 5 genes). Dose-response analysis in A549 cells (Figure 9E) evaluated IL-8 expression across a six-log range of *Aspergillus fumigatus* concentrations (0–5 × 10^6^ CFU/mL), with one-way ANOVA confirming significant dose-dependent effects (F(5,12) = 28.4, *p* < 0.0001). Time-course analysis in THP-1 macrophages (Figure 9F) tracked DEFB4A and S100A8 expression dynamics at 0, 3, 6, 12, 24, and 48 h post-stimulation, with both genes exhibiting peak expression at 24 h and distinct temporal kinetics, reflecting different upstream regulatory pathways.

## 4 Discussion

This integrated computational–experimental study provides two complementary forms of evidence for understanding pulmonary fungal infection susceptibility in patients with COPD and lung cancer. First, cross-omics integration of scRNA-seq (GSE296912) and TCGA-LUAD bulk transcriptomic data constructs and validates a random forest risk classification model achieving excellent discrimination (5-fold CV AUC = 0.988), identifying Treg infiltration, TLR4, and MMP9 as the most important predictive features. Second, transcriptomic convergence analysis identifies 79 immune-related genes shared across COPD and lung cancer transcriptomes, revealing mechanistic overlap between oncogenic pathways (EGFR, TP53, NF-κB) and antifungal immunity regulators including TLR4, S100A8, and IL-8. *In vitro* qRT-PCR validation in A549 and THP-1 cell lines confirms the biological relevance of five key candidate genes following heat-inactivated *Aspergillus fumigatus* stimulation, with macrophage-derived responses uniformly exceeding those of epithelial cells, consistent with the central role of innate myeloid immunity in antifungal defense (Pfeiffer et al., 2006; Karageorgopo et al., 2011).

The superior performance of the random forest model in this prediction task is mechanistically explicable by the inherently non-linear nature of COPD-associated fungal infection risk. COPD-related immune dysregulation involves threshold effects and complex feature interactions—particularly between PCT elevation and NLR captured by the SHAP dependence analysis—that violate the linearity assumption of logistic regression (van der Ploeg et al., 2014). Ensemble methods naturally accommodate these non-linearities, explaining their discriminative advantage. The identification of PCT, GM test, and G test as the three most influential predictors by SHAP analysis aligns with established diagnostic biomarker evidence, while the inclusion of immune function markers (CD4+/CD8+ ratio, IgG) provides novel insight into the role of systemic immune competence in fungal susceptibility (Steyerberg et al., 2013). Notably, machine learning integration of CD4^+^ T cell gene expression profiles across diverse disease contexts has demonstrated particular utility in capturing immune dysregulation signatures that transcend individual diagnostic categories, supporting the cross-disease applicability of transcriptomic feature selection approaches (Liao et al., 2025).

The *in vitro* qRT-PCR validation results provide important mechanistic insights that both support the predictive model and delineate the biological pathways governing COPD antifungal immunity. The pronounced upregulation of IL-8 (6.28-fold in A549; 8.14-fold in THP-1) reflects the critical role of this chemokine in orchestrating neutrophil recruitment to sites of Aspergillus invasion—a response that is demonstrably impaired in COPD patients receiving systemic corticosteroids (Leal et al., 2010). The strong induction of S100A8 (calprotectin) in both cell lines validates its utility as a serum biomarker of fungal infection activity in COPD. S100A8 functions as an alarmin released during neutrophil activation and macrophage fungal recognition, amplifying innate immune responses through TLR4 engagement and NF-κB activation (Cunha et al., 2012).

The use of heat-inactivated Aspergillus fumigatus conidia in the *in vitro* validation experiments warrants explicit methodological consideration. Heat inactivation was employed to ensure BSL-2 biosafety compliance and to isolate PAMP-mediated pattern recognition signaling from confounding effects of active fungal replication, metabolic activity, and secondary toxin production. This approach enables controlled interrogation of innate immune gene activation in response to fungal surface components—including β-glucan, mannan, and chitin—without the complexity of live infection dynamics. However, we acknowledge that heat inactivation alters the native PAMP profile and eliminates viability-associated immune signals (e.g., secreted proteases and gliotoxin), which may influence the magnitude and kinetics of the observed fold-changes. The generalizability of our qRT-PCR findings to live infection contexts therefore requires validation using live Aspergillus fumigatus challenge under appropriate biocontainment conditions, which represents an important direction for future work.

The amplified antifungal gene responses observed in A549 lung cancer phenotype cells have important mechanistic implications. The significantly higher induction of IL-8, S100A8, DEFB4A, MMP9, and TLR4 in lung cancer phenotype A549 cells compared to normal A549 cells following *Aspergillus fumigatus* stimulation suggests a paradoxical hyperinflammatory-yet-ineffective antifungal response phenotype. While inflammatory mediator production is amplified at the transcriptomic level, the disorganized and exhausted immune landscape characteristic of lung cancer may prevent effective fungal clearance despite elevated biomarker expression (Brand et al., 2016; Wisplinghoff et al., 2004). This dissociation between transcriptomic activation and functional antifungal immunity supports the value of machine learning models that integrate multiple immune dimensions rather than relying on single biomarker thresholds. These *in vitro* findings provide mechanistic support for the hypothesis that lung cancer represents an amplifier of IPFI susceptibility through EGFR- and TP53-mediated dysregulation of innate antifungal signaling, a hypothesis that warrants prospective clinical investigation.

The identification of EGFR as a network hub connecting oncogenic signaling to TLR4-mediated antifungal immunity provides a novel mechanistic hypothesis that deserves prospective investigation. EGFR signaling activates NF-κB and promotes IL-8 production—in lung cancer patients receiving EGFR tyrosine kinase inhibitors (TKIs), suppression of this axis may attenuate IL-8-mediated neutrophil recruitment to sites of fungal invasion (Timsit et al., 2014). The oncogenic centrality of NF-κB in promoting tumor progression through downstream inflammatory mediator cascades has been further corroborated by recent studies demonstrating that aberrant NF-κB pathway activation drives cancer metastasis across multiple histotypes, reinforcing its relevance as a shared mechanistic hub in both oncogenesis and infectious immune suppression (Zhai et al., 2025). Conversely, the negative correlation between TP53 expression and antifungal immune genes (DEFB4A and IL-8) suggests that TP53 loss-of-function mutations may suppress constitutive innate antifungal surveillance, creating a permissive microenvironment for Aspergillus colonization independent of treatment-related immunosuppression. These molecular insights provide mechanistic rationale for histotype-stratified and mutation-status-stratified IPFI prophylaxis protocols, which should be evaluated in future prospective multicenter studies.

Several limitations warrant consideration. *In vitro* cell line models, while providing controlled experimental conditions, do not fully recapitulate the complex multicellular interactions and disease-specific alterations present in COPD or lung cancer tissue. The use of heat-inactivated Aspergillus fumigatus conidia, while necessary for BSL-2 biosafety compliance, alters the native PAMP profile and may not fully recapitulate transcriptomic responses to live pathogen challenge; protein-level validation by Western blot, ELISA, or immunohistochemistry is additionally required to confirm concordance between mRNA induction and functional protein expression. The TCGA-LUAD cohort carries inherent selection bias toward surgically resectable disease, potentially underrepresenting advanced-stage and SCLC populations at highest IPFI risk. The cross-platform Pearson correlation between scRNA-seq and TCGA log_2_FC values (r = −0.088) is based on aggregate gene-level data across platforms with different normalization methods and should be interpreted as a descriptive indication of divergent disease regulation rather than as a statistically powered correlation inference. Prospective multicenter clinical validation integrating transcriptomic risk signatures with clinical biomarkers across both COPD and lung cancer populations, with histotype and mutation-status stratification, is essential before guideline integration.

## 5 Conclusion

This integrated multi-omics and machine learning study presents a validated transcriptomic risk classification framework for understanding pulmonary fungal infection susceptibility in COPD and lung cancer, achieving excellent discriminative performance (5-fold CV AUC = 0.988), with Treg infiltration, TLR4, and MMP9 as the dominant predictive features. Cross-omics integration of scRNA-seq (GSE296912) and TCGA-LUAD data identified 79 shared immune-related genes converging on NF-κB, TLR4, and cytokine receptor signaling—revealing direct molecular network connections between oncogenic hubs (EGFR, TP53, and STAT3) and antifungal immune regulators (TLR4, IL-8, DEFB4A, and S100A8). *In vitro* qRT-PCR validation confirmed significant upregulation of all five candidate genes in both A549 and THP-1 cell lines following stimulation with heat-inactivated *Aspergillus fumigatus*, with macrophage responses uniformly exceeding epithelial cell responses. Myeloid compartment expansion, NK cell cytolytic activation, and epithelial barrier disruption identified by single-cell analysis provide cellular-resolution mechanistic grounding for transcriptomic IPFI risk signatures. These findings establish a translational multi-omics framework that warrants prospective multicenter validation to enable risk-stratified antifungal management in patients with COPD and lung cancer. The broader applicability of multi-omics integration for systematically identifying oncogenic drivers and shared molecular vulnerabilities across tumor types underscores the potential of this analytical paradigm to inform precision therapeutic strategies beyond individual disease contexts (Ubaid et al., 2025).

## Data Availability

All datasets analyzed in this study are publicly available. Single-cell RNA sequencing data were obtained from the NCBI Gene Expression Omnibus under accession GSE296912 (https://www.ncbi.nlm.nih.gov/geo/query/acc.cgi?acc=GSE296912), comprising two samples: GSM8979805 (normal lung tissue, n = 3986 cells) and GSM8979808 (COPD lung tissue, n = 8092 cells). TCGA-LUAD bulk RNA-seq expression data (n = 539 tumor, n = 59 normal) and clinical annotations were retrieved from the NCI Genomic Data Commons Data Portal (https://portal.gdc.cancer.gov/). All processed data and analysis code are available in the accompanying Supplementary Materials. The qRT-PCR data generated in this study are available from the corresponding author on reasonable request.
